# Community knowledge and response to Nipah virus infection and its transmission, prevention and control measures: Insights from a cross-sectional survey in Bangladesh

**DOI:** 10.1371/journal.pntd.0013855

**Published:** 2025-12-17

**Authors:** Hemayet Hossain, Md. Abdur Nur Sakib, Snigdha Sharmin Binte Sayeed, Mostafizor Rahman, Khadiza Akter Brishty, Suzit Kumar Pal, Thofiqur Rahman, Chaw Swe Sing Marma, Md. Khairul Amin Rafi, Saiful Islam, Fahim Shahriar, Md. Shahidur Rahman Chowdhury, Md. Mahfujur Rahman

**Affiliations:** 1 Department of Anatomy and Histology, Sylhet Agricultural University, Sylhet, Bangladesh; 2 Department of Veterinary Science and Animal Husbandry, Teesta University, Rangpur, Bangladesh; 3 Faculty of Veterinary, Animal and Biomedical Sciences, Sylhet Agricultural University, Sylhet, Bangladesh; 4 Department of Microbiology and Hygiene, Bangladesh Agricultural University, Mymensingh, Bangladesh; 5 Department of Dairy and Poultry Science, Hajee Mohammad Danesh Science and Technology University, Dinajpur, Bangladesh; 6 Department of Zoology (GSSC), University of Dhaka, Dhaka, Bangladesh; 7 Faculty of Veterinary and Animal Science, Hajee Mohammad Danesh Science and Technology University, Dinajpur, Bangladesh; 8 Department of Life Sciences, Independent University, Dhaka, Bangladesh; 9 Faculty of Animal Science and Veterinary Medicine, Patuakhali Science and Technology University, Dumki, Bangladesh; 10 Department of Medicine, Sylhet Agricultural University, Sylhet, Bangladesh; Uniformed Services University: Uniformed Services University of the Health Sciences, UNITED STATES OF AMERICA

## Abstract

**Background:**

Nipah virus (NiV) is a zoonotic pathogen with high case-fatality rates, recurring outbreaks, and significant public health implications in Bangladesh, particularly in regions known as the “Nipah belt.” This study aimed to assess the knowledge, attitudes, and practices (KAP) of the general population regarding NiV transmission, prevention, and control measures.

**Methodology/principle findings:**

A cross-sectional survey was conducted from December 2024 to April 2025 among 545 respondents, selected through multi-stage sampling from 48 upazilas across 16 districts. A structured questionnaire, prepared in Bengali and English, covered four domains: sociodemographic characteristics, knowledge, attitude, and practice. Data were collected via face-to-face interviews and reported through KoboToolbox and analyzed using descriptive statistics and logistic regression. Findings revealed that only 29.2% of participants demonstrated good knowledge of NiV infection, while 94.1% showed positive attitudes toward prevention and control. However, correct preventive practices were reported by just 33.0% of respondents. Awareness of bat-to-human transmission was relatively high (66.2%), yet knowledge of the disease’s high fatality rate (11.4%) and preventive measures (28.8%) was low. Over half of participants (53.2%) reported consuming raw date palm sap, a key transmission route. Higher education, employment, and proximity to sap collection areas were associated with better knowledge, while younger age, female gender, and higher income were linked to better practices.

**Conclusion/significance:**

This first nationwide community-level KAP survey highlights critical gaps between awareness and behavior, underscoring the need for sustained, culturally tailored interventions, strengthened surveillance, and One Health approaches to reduce NiV spillover and transmission risks in Bangladesh.

## Author summary

- Only 29.2% of participants had good knowledge of NiV, with very low awareness about its high fatality rate (11.4%) and preventive measures (28.8%), despite moderate awareness of bat-to-human transmission (66.2%).
- While 94.1% of respondents showed a positive attitude toward prevention, only 33.0% reported correct preventive practices, and over half (53.2%) still consumed raw date palm sap, a major transmission route.
- Higher education, employment, and living near sap collection areas were linked to better knowledge, whereas younger age, female gender, and higher income were associated with improved preventive practices.
- Persistent raw sap consumption, driven by tradition and taste preference, indicates that awareness alone is insufficient to change risky behaviors without culturally sensitive interventions.
- Strengthen year-round NiV surveillance, integrate NiV education into school curricula, promote safer sap harvesting (e.g., bat-exclusion skirts), and implement sustained One Health-based awareness campaigns in rural and high-risk areas to bridge the gap between knowledge and practice.

## Introduction

Nipah virus (NiV) is an emerging paramyxovirus that is harbored by *Pteropus* fruit bats, which spills over to humans, causing severe neurological and respiratory disease. Human outbreaks in South and Southeast Asia are marked by exceptionally high case-fatality rates (CFR), frequently over 65% [1]. Nipah virus exhibits regular bat-to-human spillover events and has demonstrated human-to-human transmission, particularly in South-east Asia (Especially in Bangladesh and India). Due to the recognized risk, NiV is now listed by the World Health Organization (WHO) as a priority disease demanding focused R&D efforts through its Blueprint [2–4]. However, effective vaccines or targeted therapies remain unavailable [5].

Southeast Asia faces a greater risk of NiV outbreaks primarily because significant populations of *Pteropus* fruit bats, the virus’s natural reservoir which excretes the virus in saliva or urine, potentially contaminating raw date palm sap [5]. Moreover, risk is amplified by growing human contact with bats, extensive habitat loss due to deforestation, the seasonal habit of consuming raw date palm sap, and insufficient disease monitoring infrastructure in remote regions [6–8]. NiV has caused human disease outbreaks in five nations across South and Southeast Asia: Bangladesh, India, Malaysia, the Philippines, and Singapore [9]. As of May 2024, these countries collectively reported 754 laboratory-confirmed NiV infections in humans, resulting in 435 fatalities. This corresponds to an overall CFR of approximately 58%. Malaysia follows with 283 cases and 109 deaths (CFR: 39%), then India with 102 cases and 74 deaths (CFR: 73%), the Philippines with 17 cases and 9 deaths (CFR: 53%), and Singapore with 11 cases and 1 death (CFR: 9%) [9].

Bangladesh experienced its first recognized NiV outbreak in Meherpur during 2001, reporting 13 cases and nine deaths. Central and northwestern Bangladesh forms a distinct geographic zone with elevated NiV activity, known as the “Nipah belt.” Key districts within this belt include Faridpur (71 cases), Rajbari (35 cases), Naogaon (32 cases), and Lalmonirhat (24 cases) [10,11]. The virus causes remarkably high death rates, frequently surpassing 70%. This severity was starkly illustrated during the 2005 Tangail outbreak, where the fatality rate reached 92% [12]. The causative agent of NiV infection was confirmed by the CDC two years later in 2003 [13,14].

Outbreaks in Bangladesh follow a clear seasonal trend, peaking each year between December and April (winter). The risk is higher during this period due to the widespread consumption and trade of raw date palm sap, a culturally valued and economically vital product that aids transmission. This timing matches the date palm sap harvest season, which serves as a critical spillover pathway for the virus originating in infected *Pteropus* bats [15]. The disease transmits via two primary routes: consumption of contaminated, unpasteurized date palm sap, and direct person-to-person contact occurring in both household and hospital environments [16]. Bangladesh responded by establishing sentinel hospital surveillance and running public health initiatives aimed to aware people from drinking raw sap and to promote the use of bat-exclusion practices [17]. Deforestation and the resulting increase in bat-to-human contact drive recurrent NiV outbreaks in Bangladesh [18]. Compounding the issue, weak disease surveillance in rural areas allows the virus’s transmission cycle to persist, cementing its status as an endemic health threat in the region [8,12,13].

The NiV infection also represents a significant financial burden to public health both globally and in Bangladesh. In fact, the single outbreak in Malaysia during the years 1998–1999 was enormously costly, with projected damages about USD 617 million [19].

In addition, prevention measures involve measurable costs. Community behavior-change interventions in Bangladesh such as the “no raw sap” and “only safe sap” campaigns were estimated to cost approximately $30,000 and $55,000, respectively. Whereas posters and TV commercials on their own cost $96,000 and $26,000, respectively, scaling these interventions to 30 impacted districts will cost $2.6–3.5 million each season [20]. These disproportionately high effects of even minor outbreaks, such as direct medical costs, lost productivity, and additional strain on capacity within the health system, are accentuated by a lack of approved antivirals or widely available vaccines [21]. Consequently, NiV remains a significant cause of public health concern in Bangladesh and a pathogen of worldwide priority that demands stronger means of surveillance, preparedness, and prevention [9,22].

Raw date palm consumers, particularly those in high-risk areas, should be educated about the risk of Nipah virus transmission. This risk is exacerbated by inadequate surveillance systems operating at both local and global levels. To date, there is a lack of substantial research investigating the knowledge, attitudes, and practices related to the Nipah virus among the Bangladeshi population.

KAP (Knowledge, Attitude, Practice) assessment is essential for guiding effective NiV prevention and control in Bangladesh. Identifying knowledge gaps, attitudes, and actual practices helps uncover misconceptions, assess readiness to adopt preventive behaviors, and detect risky actions that facilitate spillover. These insights enable targeted health education, culturally appropriate interventions, and stronger community engagement, ultimately enhancing outbreak preparedness and reducing NiV transmission risks. Despite regular NiV outbreaks affecting both people and animals in Bangladesh; there is a paucity of in-depth studies investigating public knowledge, attitudes, and preventive practices. Previous studies mainly targeted healthcare providers and medical personnel, offering limited insight into how the general population particularly those in rural or high-risk regions perceives and responds to NiV [23,24]. Although some research identified a link between raw date palm sap consumption and NiV awareness [25], they did not explore the psychological, cultural, or economic drivers of this risky behavior, which are essential for designing effective public health interventions. Therefore, the purpose of this study was to assess public knowledge, attitudes, and practices (KAP) regarding NiV infection in Bangladesh, particularly focusing on transmission, prevention, and control measures, to identify population groups where targeted interventions are needed.

## Methods

### Ethics statement

**Fig 1:**
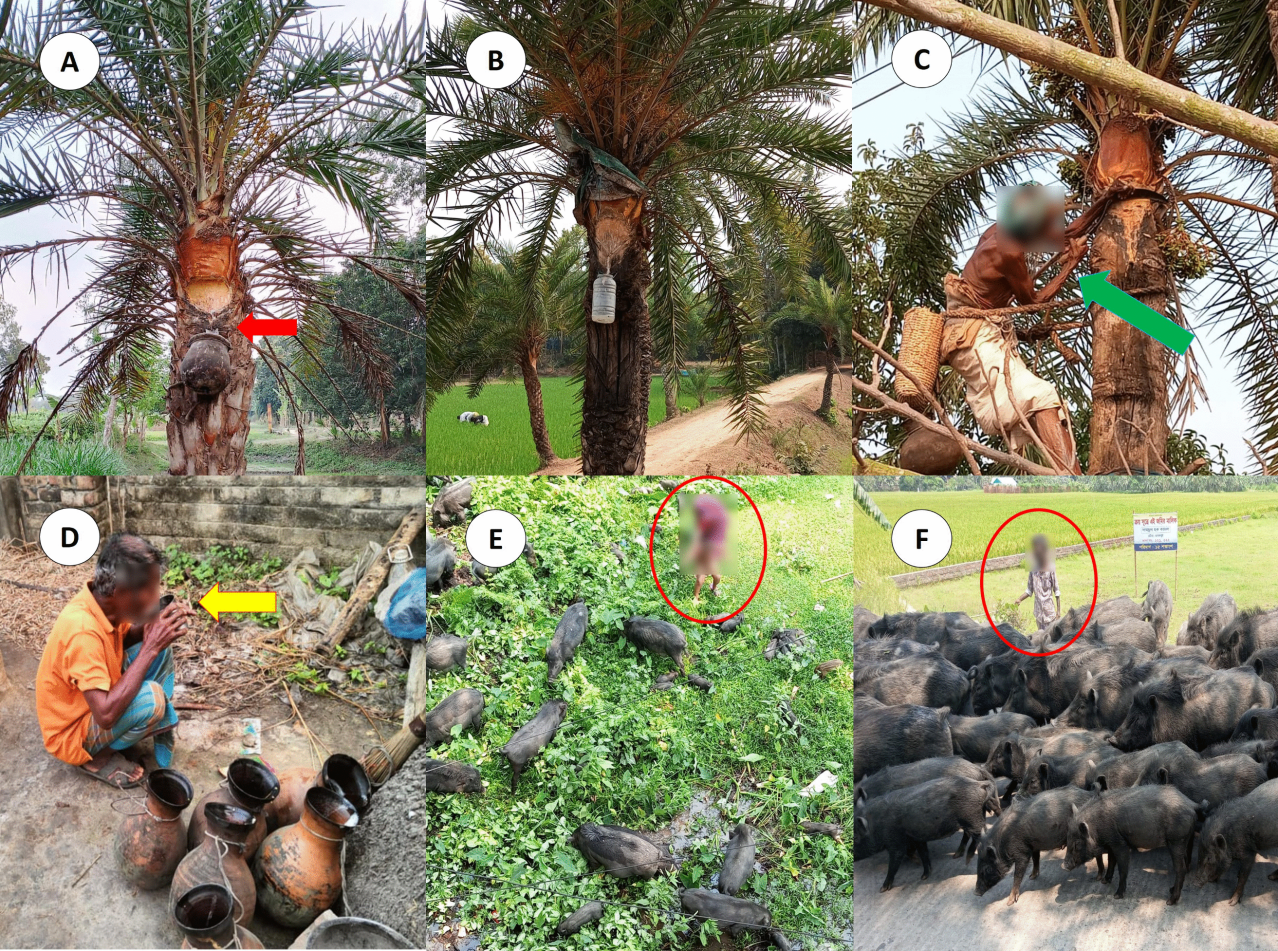
Different scenarios of raw date palm sap harvesting, handling, consumption, and human exposure to pigs. (A) Collection setup showing an unrestricted clay jar (red arrow) attached to the date palm tree for raw sap collection, allowing potential contamination by animals such as fruit bats. (B) Date palm tree prepared for sap collection with sap-flow area exposed; jar hanging without protective covering. (C) Sap harvester climbing and preparing the date palm tree for tapping (green arrow). (D) Direct consumption of untreated raw date palm sap by a person at the collection site (yellow arrow). (E) Human exposure to pigs during agricultural activities in a rural setting (red circle). (F) Large herd of pigs being tended by a person, indicating close and prolonged human–pig interaction (red circle).

Informed verbal consent was obtained from all participants prior to enrollment. Participants were informed that their participation was voluntary, their responses would remain confidential, and they could withdraw from the study at any time without penalty. They were also informed that no financial or material incentives would be provided for participation. No personal identifiers were collected in the study. The individuals who were pictured in Fig 1 has provided written informed consent (as outlined in PLOS consent form) to publish their image alongside the manuscript. This investigation was received approval through the Teesta University’s Institutional Ethics Committee, Rangpur-5404 (Ethics Approval Reference No.: TU/IEC/2025/004).

### Study design, area, and sampling

A cross-sectional survey was conducted among 545 participants using a multi-stage sampling approach from December 2024 to April 2025. Initially, the NiV belt of Bangladesh was identified based on reports from the National Institute of Health (NIH), considering factors such as the abundance of date palm trees, history of raw date palm sap consumption, selling point of raw date palm sap, exposure to pig etc. (**Fig 1**).

The identified belt included the districts of Meherpur, Naogaon, Rajbari, Faridpur, Tangail, Thakurgaon, Kushtia, Manikganj, Rajshahi, and Lalmonirhat.

**Fig 2:**
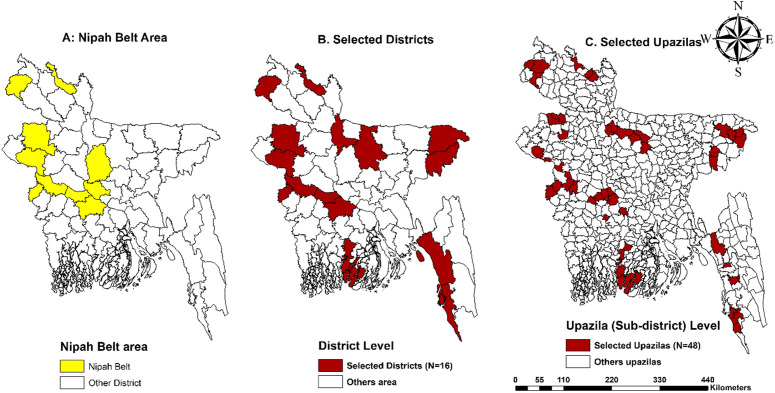
Study area map. (A) Nipah Belt Area: Map showing the Nipah belt region of Bangladesh compared to other districts. (B) District Level: Map of Bangladesh showing selected districts (n = 16) through multi-stage sampling compared with other districts (C) Upazila (Sub-district) Level: Map showing selected upazila (N = 48) compared with other upazilas. The primary shape file for the base layer of the map was extracted from GADM (https://gadm.org/) and the map was generated by ArcGIS software (ArcMap 10.8).

**Fig 3:**
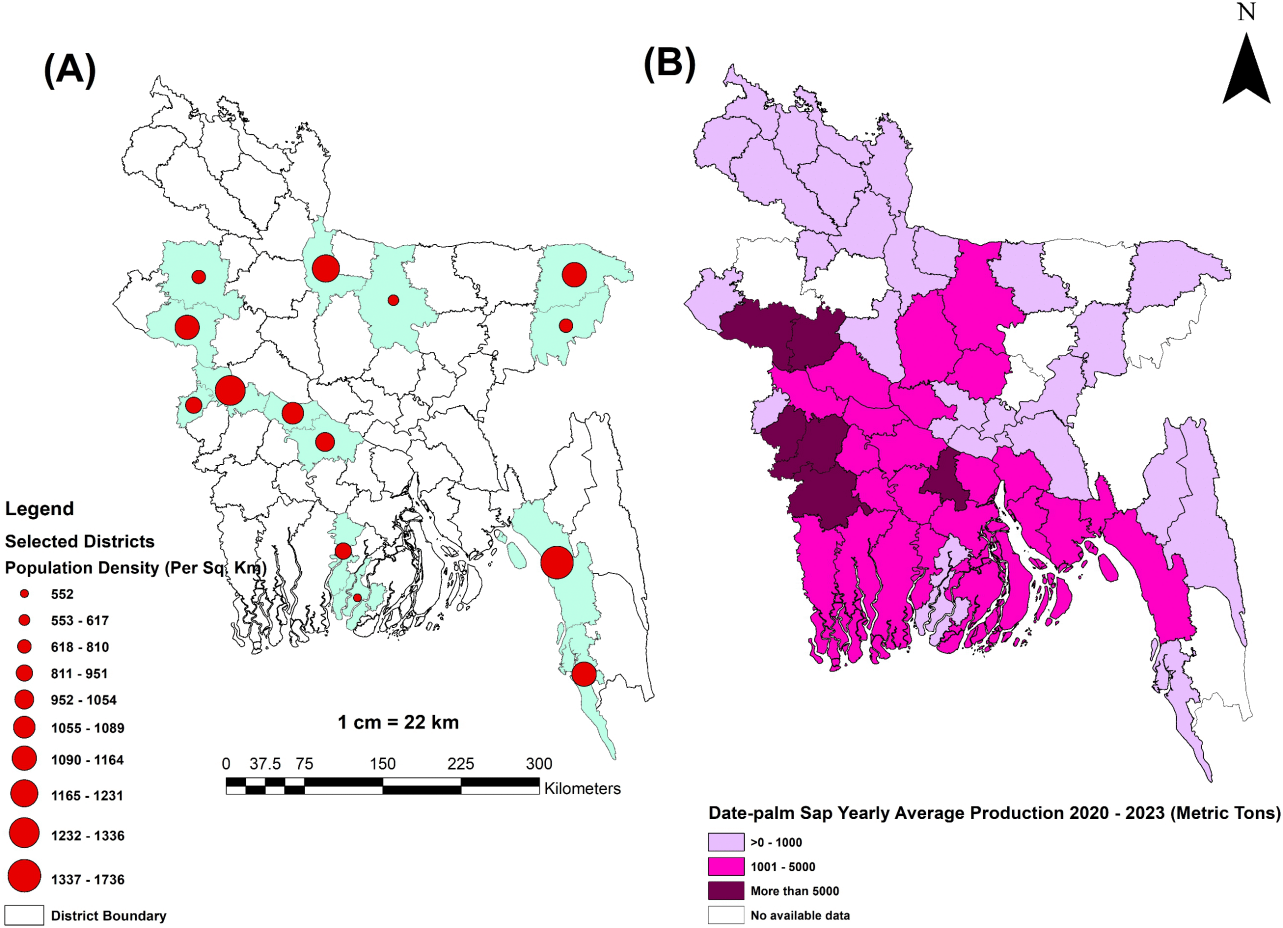
Population density and raw date palm sap production across selected districts of Bangladesh. (A) Population density of selected districts (per sq. km) is represented by the size of red circles. (B) Yearly average raw date palm sap production (2020–2023, metric tons) is shown for all districts, with color intensity representing production levels. The primary shape file for the base layer of the map was extracted from GADM (https://gadm.org/) and the map was generated by ArcGIS software (ArcMap 10.8).

From the eight administrative divisions of Bangladesh, two districts were selected from each division. From each selected district, three upazilas were chosen, resulting in a total of 16 districts and 48 upazilas (**Fig 2**). The complete list of districts and upazilas, along with their respective population densities and raw date palm production (metric tons), is provided in **Fig 3** and S1 Table. A minimum of 10 respondents were interviewed from each upazila. Within each upazila, households were systematically sampled, and one eligible participant per household was randomly selected to ensure a representative population across all regions.

The sample size was calculated using the formula described by Chow et al. [26]:

Minimum required sample, $n=\frac{Z^{2}\times Pexp\times \left(1-Pexp\right)}{d^{2}}$

[Where, n = Desired sample size; Z = 1.96 for 95% CI; P_exp_ = 0.5, Expected positive outcome (50%); d = 0.05].

Based on this calculation, the minimum required sample size was 384. However, 545 participants were ultimately surveyed to enhance statistical power and representation.

### Questionnaire design

The questionnaire was developed based on previously published literature, with necessary modifications and inputs from an expert panel [18,23,24] based on the contextual nature following etiology, transmission, epidemiology, and prevention/control measures of NiV infection for each domain. To minimize unconscious bias, questions were framed in neutral, non-leading language and reviewed by subject experts in epidemiology and infectious diseases. A pilot study was conducted to refine the questions and ensure their relevance to behavioral practices in the context of Bangladesh. Feedback from the pilot was used to refine item wording, improve clarity, and ensure that the questionnaire captured community perceptions accurately without introducing interviewer or respondent bias.

It comprised four domains (S1 Questionnaire):

**Sociodemographic profile**: 8 questions covering age, gender, education level, occupation, income, residence type, proximity to potential exposures, and healthcare access.

**Knowledge**: 11 questions addressing etiology, transmission, epidemiology, and prevention/control measures of NiV infection.

**Attitude**: 7 questions assessing participants’ perceptions and attitudes toward NiV infection and prevention.

**Practice**: 8 questions assessing preventive and control-related practice.

The questionnaire was prepared in both Bengali (local language) and English.

### Survey procedure

A pilot study involving 40 participants from the target population was carried out to assess the questionnaire’s (S1 Questionnaire) clarity, acceptability, and overall comprehensibility, ensuring its suitability for the main survey. Following the pilot testing, minor revisions were implemented to refine the questionnaire based on participant feedback and observed inconsistencies. Data obtained from the pilot trial were excluded from the final analysis. Considering the sensitive nature of NiV, data collection was carried out by trained researcher’s assistants under strict confidentiality protocols to ensure the privacy and protection of the participant information. The survey was conducted in the local language by trained enumerators. During each interview, one enumerator asked the questions while another observed and recorded the responses. Data were documented digitally using the KoboToolbox platform.

### Response to the knowledge, attitude, and practice assessment

For the knowledge assessment, a correct response was given a score of 2, while an irregular response was awarded a score of 1, and incorrect or uncertain responses received a score of 0. Attitude-based questions were measured using a five-point Likert scale: Strongly Agree (5), Agree (4), Neutral (3), Disagree (2), and Strongly Disagree (1). In practice domain, Participant’s responses were measured followed by five-point Likert scale (Always = 5, Often = 4, Sometimes = 3, Rarely = 2, Never = 1). The total knowledge, attitude, and practice (KAP) scores were calculated by summing participants’ responses within each domain, yielding score ranges of 0–22 for knowledge, 0–35 for attitude, and 0–40 for practice. The data were subsequently dichotomized into binary outcomes according to the proportion of correct responses. A 65% cut-off point was used to classify the level of knowledge, attitude, and practice. Scores equal to or above the cut-off point were categorized as good knowledge, positive attitude, and correct practices, while scores below 65% indicated poor knowledge, negative attitudes, and incorrect practices. The estimation of overall KAP levels was conducted based on previously established methods [27]. Consistent with these procedures, KAP item scores were aggregated and subsequently classified into meaningful categories using validated, established cut-off methods. This ensured uniformity in interpretation and facilitated comparison with earlier studies employing similar KAP frameworks.

### The reliability of the questionnaire

The questionnaire’s internal consistency and reliability were assessed using Cronbach’s alpha coefficient, which validated the instrument’s reliability within the context of this study. The survey instrument exhibited acceptable internal consistency and reliability, evidenced by an overall Cronbach’s alpha coefficient of 0.75. Cronbach’s alpha values above 0.60 are generally considered indicative of acceptable reliability, whereas values exceeding 0.80 reflect a high degree of internal consistency and excellent reliability [28].

### Geo-spatial mapping

The surveyed districts located within the identified Nipah virus belt were visualized in **Fig 2**. To illustrate the study area, geospatial mapping was performed using ArcMap version 10.8 (ESRI, Redlands, CA, USA). The primary shape file for the base layer of the map was extracted from GADM (https://gadm.org/). Each selected upazila (sub-district) was marked and highlighted to indicate the specific locations where data were collected.

### Statistical analysis

At the end of data collection, the complete questionnaire was manually reviewed to verify the accurate completion of variables and data quality, before being cleaned and scored using Microsoft Excel (Microsoft 365 version). All statistical analyses were conducted using R Programming (version 4.4.2). Reproducible descriptive tables, univariate and multivariate logistic regression tables, were created using the gtsummary-R package for reproducible research [29].

Descriptive statistics, including frequencies and percentages, were employed to summarize categorical variables. Univariable and multivariable analyses were conducted to assess the associations between sociodemographic variables (independent factors) and Knowledge, Attitudes, and Practices (KAP) outcomes (dependent factors), using a significance level of *P 0.05*. Univariable logistic regression analysis was employed to estimate odds ratios (ORs) and corresponding 95% confidence intervals (CIs) for various sociodemographic variables. To achieve comprehensive adjustment, all sociodemographic factors were incorporated into the multivariable logistic regression model, irrespective of their significance in the univariate analysis. A multivariable logistic regression model was used to estimate adjusted odds ratios (AORs) along with their corresponding 95% confidence intervals (CIs). Statistical significance was defined as a p-value less than 0.05, and results were reported as adjusted odds ratios (AORs) accompanied by 95% confidence intervals (CIs). Furthermore, Spearman’s rank correlation coefficient was utilized to assess the relationships among knowledge, attitudes, and practices.

## Results

### Sociodemographic characteristics of the participants

A total of 545 individuals participated in the study (**Table 1**). The majority were aged between 18–25 years (43.8%), followed by those aged 26–35 years (29.4%) and 36–45 years (11.2%).

**Table 1:** Sociodemographic characteristics of participants, N = 545.

Variables	Frequency (n)	Percentage (%)	95% CI
**Age of participants (Years)**			
13–18	24	4.4	2.8–6.5
18–25	239	43.8	39.6–48.1
26–35	160	29.4	25.6–33.4
36–45	61	11.2	8.7–14.1
45 above	61	11.2	8.7–14.1
**Biological gender of the participant**			
Female	122	22.4	19.0–26.1
Male	423	77.6	73.9–81.1
**Education level**			
No or primary education	63	11.6	9.0–14.6
Secondary	59	10.8	8.3–13.7
Higher secondary	111	20.4	17.1–24.0
Tertiary (bachelor’s & above)	312	57.2	53.0–61.4
**Participant type based on occupation**			
Student	276	50.6	46.4–54.9
Homemakers	52	9.5	7.2–12.3
Manual Labor & Unemployed	68	12.5	9.8–15.6
Service Holders (Government & Private)	104	19.1	15.9–22.6
Business & Self-employed	45	8.3	6.1–10.9
**Income level (BDT)**			
No income	257	47.1	42.9–51.4
Below 10000	106	19.5	16.2–23.0
10001–30000	105	19.3	16.0–22.8
Above 30000	45	14.1	11.3–17.3
**Type of Participants living area**			
Peri-urban/ Semi-urban	119	21.8	18.4–25.5
Rural	199	36.5	32.5–40.7
Rural	227	41.7	37.5–45.9
**Proximity to exposures of raw date palm sap (Square km)**			
Less than 5	251	46.1	41.8–50.3
5-10	114	20.9	17.6–24.6
> 10	180	33.0	29.1–37.2
**Near healthcare access**			
1–5 km	373	68.4	64.4–72.3
6–10 km	100	18.4	15.2–21.9
More than 10 km	72	13.2	10.5–16.4

Males comprised a significant portion of the participants (77.6%), with females accounting for 22.4%. Regarding educational attainment, 57.2% had tertiary-level education, while only 11.6% had no or primary-level education. In terms of occupation, students constituted the largest group (50.6%), followed by service holders (19.1%) and manual laborers or the unemployed (12.5%). Almost half of the participants (47.1%) reported having no income mostly are university students. A considerable proportion (36.5%) were residents of rural areas. Notably, 46.1% of participants lived within five square kilometers of raw date palm sap exposure zones, potentially heightening their exposure risk. Most respondents (68.4%) reported having access to healthcare within 1–5 kilometers from their residence (**Table 1**).

### Knowledge, attitude, and practice (KAP) outcomes

#### Knowledge.

Only 159 (29.2%) respondents demonstrated good knowledge about NiV, whereas 386 (70.8%) exhibited poor knowledge (**Table 2**).

**Table 2:** Overall KAP scores of the participants (N = 545).

Level of measures	KAP Scores		
	Frequency (n)	Percentage (%)	95% CI
**Knowledge outcome**			
Good	159	29.2	25.4, 33.2
Poor	386	70.8	66.8, 74.6
**Attitude outcome**			
Positive	513	94.1	91.8, 96.0
Negative	32	5.9	4.1, 8.2
**Practice outcome**			
Correct	180	33.0	29.1, 37.2
Incorrect	365	67.0	62.9, 70.9

**Fig 4:**
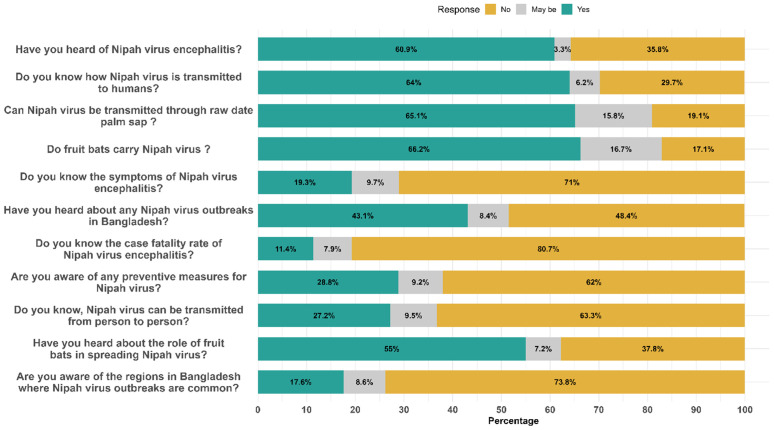
Knowledge level of participants regarding NiV infection, epidemiology and its control.

Item-level analysis from S2 Table reveals specific knowledge gaps. Although a large majority had heard of NiV infection (60.9%) and were aware of bat-to-human transmission (66.2%), only 11.4% knew about the high case fatality rate of the disease (**Fig 4**). Moreover, a mere 28.8% were aware of any preventive measures, and only 27.2% knew that person-to-person transmission was possible. Alarmingly, 71.0% of respondents could not identify symptoms of NiV infection, and 73.8% were unaware of the geographical hotspots in Bangladesh where outbreaks are common. Interestingly, 53.2% admitted to usually drinking raw date palm sap, indicating a significant behavioral risk despite moderate awareness of transmission routes (e.g., 65.1% affirmed NiV transmission through raw sap) (**Fig 4**).

#### Attitude.

**Fig 5:**
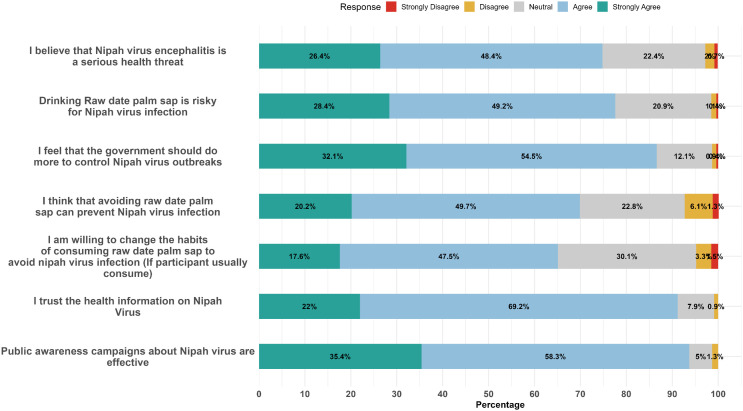
Attitude level of participants regarding Nipah virus infection and its control.

Despite the generally poor knowledge levels, the attitude toward NiV prevention and control was largely positive, with 513 (94.1%) participants showing a favorable disposition (**Table 2**). As indicated in S3 Table, most participants (n = 408) recognized NiV as a serious health threat (74.8% agreed/strongly agreed). Approximately, 423 (77.6%) participants agreed that drinking raw date palm sap is risky, and 81.6% believed that the government should do more to control NiV outbreaks. Furthermore, a substantial number (65.1%) agreed or strongly agreed that they were willing to change raw sap consumption habits to avoid infection, although neutrality and reluctance were also present (30.1%). Trust in public health messaging was high (91.2% agreed/strongly agreed), and 93.7% considered awareness campaigns effective (**Fig 5).**

#### Practice.

**Fig 6:**
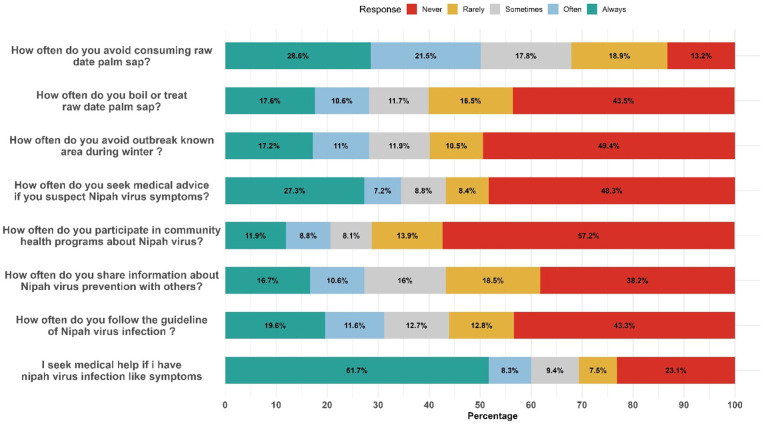
Practice level of participants regarding NiV infection and its control.

Contrasting with positive attitudes, practical behaviors lagged behind: 67.0% of participants reported incorrect practices (**Table 2**). According to S4 Table, while 28.6% always avoided raw date palm sap, 32.1% either rarely or never did. Boiling or treating raw date palm sap (RDPS) was infrequent only 28.2% did so often or always. Avoidance of known outbreak areas during winter was uncommon (only 28.2% always/often avoided), and health-seeking behavior was suboptimal only 34.5% reported they would always or often seek medical advice when experiencing symptoms. Participation in community health programs was notably low (57.2% never participated), and only 27.3% always or often shared preventive information. While 51.7% claimed they would always seek medical help upon experiencing symptoms, guideline adherence remained poor: 43.3% never followed NiV-related recommendations (**Fig 6**).

### Factors associated with knowledge level

Univariate and multivariable logistic regression analysis identified several significant predictors of knowledge regarding NiV infection (**Table 3**).

**Table 3:** Univariable and multivariable analyses show the association of demographic factors with the knowledge level, N = 545.

Variables	Knowledge level		Univariate analysis			Multivariate analysis		
	Poor (N = 386)	Good (N = 159)	OR	95% CI	*p*-value	AOR	95% CI	*p*-value
**Age of Participants**								
13–18	23 (6.0%)	1 (0.6%)	*Ref.*	—		—	—	
18–25	156 (40%)	83 (52.2%)	12.2	2.51, 221	0.015	4.05	0.63, 80.9	0.2
26–35	99 (26%)	61 (38.4%)	14.2	2.87, 257	0.010	4.07	0.60, 83.1	0.2
36–45	54 (14%)	7 (4.4%)	2.98	0.49, 57.4	0.3	1.47	0.17, 32.4	0.8
45 above	54 (14%)	7 (4.4%)	2.98	0.49, 57.4	0.3	1.86	0.23, 40.7	0.6
**Biological gender of the participant**								
Female	92 (24%)	30 (19%)	*Ref.*	—		—	—	
Male	294 (76%)	129 (81%)	1.35	0.86, 2.16	0.2	0.90	0.50, 1.61	0.7
**Education level**								
No or Primary Education	60 (15.5%)	3 (1.9%)	*Ref.*	—		—	—	
Secondary	54 (14%)	5 (3.1%)	1.85	0.43, 9.37	0.4	2.23	0.48, 12.1	0.3
Higher Secondary	88 (22.7%)	23 (14%)	5.23	1.72, 22.7	0.009	3.43	0.95, 16.6	0.083
Tertiary (bachelor’s & above)	184 (47.8%)	128 (81%)	13.9	5.01, 57.8	<0.001	7.71	2.20, 36.8	0.003
**Participant type based on occupation**								
Homemakers	47 (12%)	5 (3.1%)	*Ref*.	—		—	—	
Student	186 (48%)	90 (56.8%)	4.55	1.91, 13.5	0.002	1.73	0.54, 6.38	0.4
Manual Labor & Unemployed	58 (15%)	10 (6.3%)	1.62	0.54, 5.50	0.4	1.71	0.44, 7.20	0.4
Service Holders (Government & Private)	56 (15%)	48 (30%)	8.06	3.21, 24.7	<0.001	5.89	1.38, 28.7	0.021
Business & Self-employed	39 (10%)	6 (3.8%)	1.45	0.41, 5.36	0.6	2.17	0.43, 11.3	0.3
**Income level**								
10001–30000	80 (21%)	25 (16%)	*Ref.*	—		—	—	
No income	182 (47%)	75 (47%)	1.32	0.79, 2.26	0.3	2.68	0.97, 7.99	0.065
Below 10000	76 (20%)	30 (19%)	1.26	0.68, 2.35	0.5	2.34	0.88, 6.66	0.10
Above 30000	48 (12%)	29 (18%)	1.93	1.02, 3.70	0.045	1.63	0.74, 3.63	0.2
**Type of Participants living area**								
Urban	163 (42%)	64 (40.3%)	*Ref.*	—		—	—	
Rural	141 (37%)	58 (36.4%)	1.05	0.69, 1.60	0.8	1.35	0.78, 2.32	0.3
Peri-urban/ Semi-urban	82 (21%)	37 (23.3%)	1.15	0.70, 1.86	0.6	1.12	0.65, 1.91	0.7
**Proximity to exposures of raw date palm sap**								
> 10 km	138 (35.7%)	42 (26.5%)	*Ref*.	—		—	—	
Less than 5 sq km	165 (42.7%)	86 (54%)	1.71	1.12, 2.66	0.015	2.30	1.41, 3.82	0.001
5–10 km	83 (21.6%)	31 (19.5%)	1.23	0.71, 2.10	0.5	1.37	0.75, 2.47	0.3
**Near healthcare access**								
6–10 km	71 (18.4%)	29 (18%)	*Ref.*	—		—	—	
1–5 km	264 (68.4%)	109 (69%)	1.01	0.63, 1.66	>0.9	0.97	0.55, 1.75	>0.9
More than 10 km	51 (13.2%)	21 (13%)	1.01	0.51, 1.96	>0.9	1.08	0.50, 2.34	0.8

OR: Odds Ratio, AOR: Adjusted Odds Ratio, CI: Confidence Interval.

Participants with tertiary education had markedly better knowledge compared to those with no or primary education (AOR: 7.71, 95% CI: 2.20–36.8, *p* = 0.003). Employment also played a role; service holders were significantly more likely to have good knowledge (AOR: 5.89, 95% CI: 1.38–28.7, *p* = 0.021). Additionally, living within five kilometers of raw date palm sap exposure areas was positively associated with good knowledge (AOR: 2.30, 95% CI: 1.41–3.82, *p* = 0.001), likely due to increased exposure and awareness of localized risk factors (**Table 3).**

### Factors associated with attitude level

Educational level was a significant determinant of attitude. Participants with tertiary education (AOR: 5.34, 95% CI: 1.15–25.9, *p* = 0.034) and higher secondary education (AOR: 4.93, 95% CI: 1.14–24.0, *p* = 0.038) had more favorable attitudes. Students, in particular, were significantly associated with positive attitudes (AOR: 13.7, 95% CI: 2.27–87.8, *p* = 0.005), possibly reflecting the impact of academic environments on health awareness. Participants residing in peri-urban or semi-urban areas had significantly higher odds of having a positive attitude (AOR: 4.15, 95% CI: 1.16–20.4, *p* = 0.046). Moreover, living within 5–10 kilometers of raw sap exposure areas was associated with a more positive attitude (AOR: 8.24, 95% CI: 1.82–63.0, *p* = 0.015), possibly indicating indirect exposure to risk messaging and preventive campaigns in neighboring areas (**Table 4**).

**Table 4:** Univariable and multivariable analyses show the association of demographic factors with the attitude level, N = 545.

Variables	Attitude Level		Univariate analysis			Multivariate analysis		
	Negative (N = 32)	Positive (N = 513)	OR	95% CI	*p*-value	AOR	95% CI	*p*-value
**Age of Participants**								
13–18	5 (15.6%)	19 (3.7%)	*Ref.*	—		—	—	
18–25	11 (34.3%)	228 (44.3%)	5.45	1.59, 16.8	0.004	1.03	0.18, 5.59	>0.9
26–35	4 (12.5%)	156 (30%)	10.3	2.52, 44.7	0.001	5.34	0.65, 46.5	0.12
36–45	7 (21.9%)	54 (11%)	2.03	0.55, 7.14	0.3	2.46	0.31, 18.7	0.4
45 above	5 (15.7%)	56 (11%)	2.95	0.75, 11.7	0.12	5.85	0.77, 46.2	0.088
**Biological gender of the participant**								
Female	8 (25%)	114 (22%)	*Ref.*	—		—	—	
Male	24 (75%)	399 (78%)	1.17	0.48, 2.56	0.7	0.46	0.09, 1.78	0.3
**Education level**								
No or Primary Education	10 (31%)	53 (10.3%)	*Ref.*	—		—	—	
Secondary	9 (28%)	50 (9.7%)	1.05	0.39, 2.84	>0.9	1.13	0.34, 3.77	0.8
Higher Secondary	5 (16%)	106 (21%)	4.00	1.35, 13.4	0.016	4.93	1.14, 24.0	0.038
Tertiary (bachelor’s & above)	8 (25%)	304 (59%)	7.17	2.71, 19.6	<0.001	5.34	1.15, 25.9	0.034
**Participant type based on occupation**								
Business & Self-employed	8 (25%)	37 (7.2%)	*Ref.*	—		—	—	
Student	10 (31.2%)	266 (52%)	5.75	2.08, 15.5	<0.001	13.7	2.27, 87.8	0.005
Homemakers	7 (21.9%)	45 (8.8%)	1.39	0.46, 4.31	0.6	0.88	0.11, 6.74	0.9
Manual Labor & Unemployed	5 (15.6%)	63 (12%)	2.72	0.85, 9.60	0.10	3.43	0.80, 15.9	0.10
Service Holders (Government & Private)	2 (6.3%)	102 (20%)	11.0	2.62, 75.3	0.003	5.33	0.90, 44.6	0.081
**Income level**								
Below 10000	8 (25%)	98 (19.1%)	*Ref.*	—		—	—	
No income	14 (43.8%)	243 (47.4%)	1.42	0.55, 3.42	0.4	0.86	0.23, 2.85	0.8
10001–30000	7 (21.9%)	98 (19.1%)	1.14	0.40, 3.38	0.8	1.17	0.29, 4.77	0.8
Above 30000	3 (9.3%)	74 (14.4%)	2.01	0.56, 9.43	0.3	1.15	0.18, 8.30	0.9
**Type of Participants living area**								
Urban	18 (56.2%)	209 (40.7%)	*Ref.*	—		—	—	
Rural	11 (34.4%)	188 (36.6%)	1.47	0.69, 3.29	0.3	2.87	0.91, 9.80	0.080
Peri-urban/ Semi-urban	3 (9.4%)	116 (22.7%)	3.33	1.10, 14.4	0.058	4.15	1.16, 20.4	0.046
**Proximity to exposures of raw date palm sap**								
> 10 km	15 (46.8%)	165 (32%)	*Ref.*	—		—	—	
Less than 5 sq km	15 (46.8%)	236 (46%)	1.43	0.68, 3.03	0.3	2.12	0.83, 5.62	0.12
5–10 km	2 (6.4%)	112 (22%)	5.09	1.40, 32.7	0.033	8.24	1.82, 63.0	0.015
**Near healthcare access**								
More than 10 km	6 (18.7%)	66 (12.8%)	*Ref.*	—		—	—	
1–5 km	21 (65.6%)	352 (68.6%)	1.52	0.54, 3.71	0.4	1.74	0.46, 5.97	0.4
6–10 km	5 (15.7%)	95 (18.6%)	1.73	0.50, 6.22	0.4	1.68	0.39, 7.44	0.5

### Factors associated with practice level

Several factors significantly influenced correct preventive practices (**Table 5**). Younger age groups, specifically 13–18 years (AOR: 7.97, 95% CI: 2.12–29.9, *p* = 0.002) and 18–25 years (AOR: 3.09, 95% CI: 1.31–7.53, *p* = 0.011), were more likely to follow correct practices, potentially due to better health literacy. Females were more compliant with preventive measures compared to males (AOR: 2.34, 95% CI: 1.33–4.14, *p* = 0.003). Occupation also influenced behavior service holders (AOR: 3.02, 95% CI: 1.23–7.78, *p* = 0.018) and manual laborers/unemployed individuals (AOR: 2.75, 95% CI: 1.06–7.55, *p* = 0.042) had higher odds of correct practices.

**Table 5:** Univariable and multivariable analyses show the association of demographic factors with the practice level, N = 545.

Characteristic	Practice level		Univariate analysis			Multivariate analysis		
	Incorrect (N = 365)	Correct (N = 180)	OR	95% CI	*p*-value	AOR	95% CI	*p*-value
**Age of Participants**								
36–45	43 (11.8%)	18 (10%)	*Ref.*	—		—	—	
13–18	14 (3.9%)	10 (5.6%)	1.71	0.63, 4.55	0.3	7.97	2.12, 29.9	0.002
18–25	168 (46%)	71 (39.5%)	1.01	0.55, 1.90	>0.9	3.09	1.31, 7.53	0.011
26–35	108 (29.6%)	52 (28.8%)	1.15	0.61, 2.22	0.7	1.33	0.63, 2.86	0.5
45 above	32 (8.7%)	29 (16.1%)	2.16	1.04, 4.62	0.042	2.41	1.07, 5.53	0.035
**Biological gender of the participant**								
Male	293 (80%)	130 (72%)	*Ref.*	—		—	—	
Female	72 (20%)	50 (28%)	1.57	1.03, 2.37	0.035	2.34	1.33, 4.14	0.003
**Education level**								
Higher Secondary	85 (23.2%)	26 (14.4%)	*Ref.*	—		—	—	
No or Primary Education	43 (11.8%)	20 (11.1%)	1.52	0.76, 3.03	0.2	0.86	0.36, 2.00	0.7
Secondary	35 (9.6%)	24 (13.3%)	2.24	1.14, 4.45	0.020	1.37	0.58, 3.21	0.5
Tertiary (bachelor’s & above)	202 (55.4%)	110 (61.2%)	1.78	1.10, 2.97	0.023	1.46	0.83, 2.64	0.2
**Participant type based on occupation**								
Business & Self-employed	34 (9.4%)	11 (6%)	*Ref.*	—		—	—	
Homemakers	33 (9.0%)	19 (11%)	1.78	0.74, 4.41	0.2	1.22	0.35, 4.35	0.8
Manual Labor & Unemployed	41 (11.2%)	27 (15%)	2.04	0.90, 4.83	0.10	2.75	1.06, 7.55	0.042
Service Holders (Government & Privet)	52 (14.2%)	52 (29%)	3.09	1.45, 7.00	0.005	3.02	1.23, 7.78	0.018
Student	205 (56.2%)	71 (39%)	1.07	0.53, 2.32	0.9	0.86	0.30, 2.58	0.8
**Income level**								
No income	187 (51.1%)	70 (39%)	*Ref.*	—		—	—	
Below 10000	71 (19.5%)	35 (19%)	1.32	0.80, 2.14	0.3	1.50	0.85, 2.64	0.2
10001–30000	71 (19.5%)	34 (19%)	1.28	0.78, 2.09	0.3	1.35	0.57, 3.15	0.5
Above 30000	36 (9.9%)	41 (23%)	3.04	1.80, 5.17	<0.001	2.78	1.02, 7.61	0.045
**Type of Participants living area**								
Urban	161 (44%)	66 (37%)	*Ref.*	—		—	—	
Rural	134 (37%)	65 (36%)	1.18	0.78, 1.79	0.4	0.70	0.40, 1.21	0.2
Peri-urban/ Semi-urban	70 (19%)	49 (27%)	1.71	1.07, 2.72	0.024	1.47	0.87, 2.48	0.15
**Proximity to exposures of raw date palm sap**								
> 10 km	132 (36%)	48 (26.7%)	*Ref.*	—		—	—	
5–10 km	73 (20%)	41 (22.8%)	1.54	0.93, 2.56	0.092	2.00	1.12, 3.59	0.020
Less than 5 sq km	160 (44%)	91 (50.5%)	1.56	1.03, 2.39	0.036	2.06	1.26, 3.42	0.004
**Near healthcare access**								
1–5 km	271 (74%)	102 (57%)	*Ref.*	—		—	—	
6–10 km	58 (16%)	42 (23%)	1.92	1.21, 3.04	0.005	1.86	1.10, 3.16	0.021
More than 10 km	36 (10%)	36 (20%)	2.66	1.59, 4.46	<0.001	3.86	2.08, 7.26	<0.001

OR: Odds Ratio, AOR: Adjusted Odds Ratio, CI: Confidence Interval.

Income level was another important factor; individuals earning more than 30,000 BDT were significantly more likely to engage in proper practices (AOR: 2.78, 95% CI: 1.02–7.61, *p* = 0.045). Proximity to raw date palm sap exposure within five kilometers (AOR: 2.06, 95% CI: 1.26–3.42, *p* = 0.004) and 5–10 kilometers (AOR: 2.00, 95% CI: 1.12–3.59, *p* = 0.020) was also positively associated. Notably, living more than 10 kilometers from healthcare services significantly increased the odds of correct practices (AOR: 3.86, 95% CI: 2.08–7.26, *p* < 0.001), possibly indicating self-reliant or preventive behavior in areas with limited access to professional care.

#### Correlation among knowledge, attitude, and practice.

Spearman’s rank correlation analysis revealed a weak but statistically significant positive correlation between knowledge and attitude (*ρ* = 0.143, *p* = 0.001) and between knowledge and practice (*ρ* = 0.219, *p* < 0.001) as depicted in **Table 6**. However, the correlation between attitude and practice was not statistically significant (*ρ* = 0.076, *p* = 0.077). These findings suggest that while knowledge may modestly influence attitude and practice, attitude alone does not necessarily predict preventive behavior, indicating a gap between awareness, perception, and behavioral implementation.

**Table 6:** Spearman’s rank correlation among respondents of knowledge, attitude, and practice.

Association	Correlation (*ρ*)	*P* value
Knowledge and Attitudes	0.143	0.001
Knowledge and Practices	0.219	<0.001
Attitudes and Practices	0.076	0.077

## Discussion

This study provides critical insights into the public’s knowledge, attitudes, and practices (KAP) concerning NiV infection in the Nipah belt and other regions susceptible to outbreaks of Bangladesh. Despite a moderately high level of disease awareness, the findings reveal substantial deficiencies in knowledge and preventive behaviors among the general population.

Specifically, only 29.2% of respondents demonstrated good knowledge about NiV infection. However, a strong positive attitude toward NiV prevention and control was observed in 94.1% of participants. In contrast, only 33.0% reported engaging in appropriate preventive practices. These findings underscore a significant disconnect between awareness, perception, and actual behavior, pointing to the need for tailored, community-centered health education and behavior change strategies.

Bangladesh faces heightened NiV risk because winter (December–April) coincides with the peak collection and consumption of raw date palm sap, a culturally favored beverage that is frequently contaminated by *Pteropus* bats, the reservoir for NiV [15]. Seasonal cooling may also prolong viral survival in sap, further elevating spillover risk [30]. Despite repeated outbreaks, control and prevention remain challenging: consumption of untreated raw sap persists by preference and habit, and risk communications have struggled to shift behavior at scale [31]. While Bangladesh operates sentinel surveillance, attention and resources often intensify only during the short outbreak season, limiting sustained, year-round risk reduction [12].

*Pteropus* bats play a central role as the primary reservoir responsible for NiV (Clade I: NiV-B) outbreaks, whereas in countries such as Malaysia, Singapore, and the Philippines, outbreaks have been linked predominantly to Clade II (NiV-M) [9] and were driven primarily by pathogen spillover through infected pigs, horse to human [32]. In Bangladesh and the neighboring region of India, the case fatality rate remains drastically higher than in other affected countries. The major drivers in Bangladesh and India include the close human–bat interface and the widespread consumption of raw date palm sap, which facilitates viral spillover [22]. As highlighted by Khan et al. (2024), these factors underscore the urgent need for government-led interventions focusing on limiting access of bats to sap collection sites, promoting safe sap consumption practices, and addressing ecological drivers such as deforestation that disrupt bat habitats and increase the likelihood of spillover events [9].

Geographically, many high-risk districts lie within the “Nipah belt,” where bat roosts, abundant date palm trees, and widespread sap harvesting create frequent human bat interface [30]. Person-to-person transmission, especially via respiratory secretions can amplify outbreaks in dense communities and overstretched hospitals, raising the threat of larger clusters [33]. In the absence of consistent prevention strategies, including bamboo skirt use to block bat access to sap, along with strong risk communication, Bangladesh could continue to experience recurrent and lethal NiV epidemics, given the virus’s high mortality and lack of targeted therapies or vaccines [6,25].

According to a previous study, 50% of respondents had a moderate level of knowledge about the NiV among doctors, including prevention [23]. As this study targeted the general population, who have less disease-specific knowledge than healthcare professionals, it reported a lower level of knowledge. Lack of knowledge among the population serves as a barrier to the early diagnosis and treatment of zoonotic infections [34]. The same reason is valid for another study on healthcare workers (HCWs), where a higher good knowledge level (46.15%) was recorded than our study [24]. Moreover, only 37% of participants in a study [25], which was conducted the survey in two endemic regions of Bangladesh (Rajbari and Kushtia) stated that they had heard of Nipah through the intake of raw sap. On the other hand, 65.1% of their participants were aware of the disease. This difference could likely be attributed to the variety of respondents, the majority of whom were students. The other possible reason is that people heard about NiV infection from various sources such as campaigns, awareness programs, social media, television and academic institutions. Lastly, a study reported the first efforts to measure the public knowledge on the national level [18]; however, this survey included the level of attitude and practice as well. In another study conducted on NiV infection in South India, shown that 77.8% of medical staff were aware of NiV symptoms whereas 95% also correctly recognized the primary reservoir as fruit bats [35]. Though for religious purpose people avoid pig consumption which work for reducing spread of NiV in this country as pig is reservoir host for Nipah. Moreover, a Malaysian KAP investigation found notably higher levels of knowledge (71%), attitude (99%), and practice (64%) concerning diseases transmitted by bats [36]. Differences in context among countries might influence the relatively lower outcomes of this study. However, in a study conducted in the Kingdom of Saudi Arabia, healthcare workers had adequate knowledge (45%), exceeding the general population although less than that of Bangladeshi healthcare workers [37]. Similarly, following an Indian study, 37% of rural participants believed they were at risk of contracting NiV [38].

This study calls for a more integrated and interdisciplinary approach to the prevention and management of Nipah virus infection in Bangladesh; thus, it requires a strong One Health plan. The health system alone cannot adequately address Nipah virus, a neglected zoonotic disease emerging at the human–animal–environment interface. Meurer, (2025) emphasized that successful control of zoonoses demands coordinated efforts across the human, veterinary, and environmental sectors to address not only knowledge gaps but also to improve surveillance and response systems [34]. Enhanced inter-sectoral communication, increased laboratory capacity, and integrated surveillance networks for rapidly identifying infections are needed [9,34]. This is very concerning in Bangladesh, where outbreaks investigation of Nipah are usually performed only after human cases come to attention.

Despite positive attitudes, 53.2% of people continue to consume raw date sap as a result of traditional beliefs in the health and purity of raw date sap; this allows traders the incentive to continue dangerous practices and spread NiV infection. In rural communities, sap collection and consumption represent not only a dietary preference but also a seasonal livelihood. So, making abrupt changes on their natural habit as preventive control will be difficult without viable economic or socially acceptable alternatives.

Collectively, the evidence demonstrates that improving KAP related to NiV infection requires a multi-layered “One Health” approach that integrates continuous community engagement, locally adapted educational materials, improved surveillance, vector control and supportive policies that encourage safer sap collection and consumption methods. Without such integrated efforts, behavioral gaps are likely to persist, particularly in regions where traditional practices and environmental conditions consistently favor spillover dynamics.

### Strengths and limitations

The present study, the first nationwide community-level KAP study on Nipah Virus infection in Bangladesh, emerges considering the threat of Nipah Virus outbreaks and its emergence as a zoonotic threat from deforestation and raw sap consumption. Notably, the survey utilized a closed-ended questionnaire, which may have introduced response bias, even though the data were collected through a self-administered format. In addition, the assessment of knowledge, attitudes, and practices was conducted using a limited set of survey items. The robust multi-stage sampling, large sample size, and use of a validated questionnaire enhance the reliability and generalizability of findings. Data collection during the active outbreak season adds contextual relevance. As with any cross-sectional study, causal inferences cannot be drawn, and self-reported responses may be subject to recall or social desirability bias; however, these are unlikely to significantly affect the overall conclusions. Despite these limitations, this exploratory study provides valuable insights that can inform public health authorities in Bangladesh and guide the design of future Nipah virus infection awareness campaigns and intervention strategies. Further research is warranted to better understand the risks associated with Nipah virus and to inform the development of effective policies aimed at improving both animal and human health outcomes.

## Conclusion

This nationwide community-based survey reveals a critical imbalance between the Bangladeshi population’s awareness of Nipah Virus and their actual preventive behaviors. While the majority of respondents demonstrated positive attitudes toward Nipah Virus prevention and control, less than one-third possessed good knowledge, and only one-third adhered to correct preventive practices. Persistent consumption of raw date palm sap, despite awareness of its risk, highlights deep-rooted cultural habits and insufficient translation of knowledge into behavior change. Given the high fatality rate of Nipah Virus, seasonal clustering of outbreaks in the Nipah belt, and ongoing ecological drivers such as deforestation and bat human contact, these findings underscore the urgent need for targeted, sustained interventions. Public health policies should focus on strengthening surveillance systems, enhancing syndromic reporting, and implementing continuous awareness campaigns particularly in rural and high-risk communities. Integrating Nipah Virus education into school curricula, promoting safer sap harvesting methods, and leveraging culturally sensitive communication strategies can foster more sustainable behavioral change. Implementing strategies such as community education and awareness program on safe fruit consumption, reducing raw date palm sap intake, and monitoring bat-human interactions within a One Health framework through inter-sectoral collaboration among public health, veterinary, and wildlife authorities could significantly reduce the risk of future Nipah virus (NiV) outbreaks in Bangladesh.

### Recommendation

The findings of this study suggest that public health policy should prioritize strengthening surveillance, syndromic reporting, and public health preparedness in the Nipah belt. Moreover, to integrate disease, human behavior, and wildlife, the study supports a One Health approach by encouraging safer sap harvesting and culturally sensitive health communication, including the active involvement of local health leaders, as well as region-specific education initiatives such as incorporating Nipah Virus infection into school health curricula or community awareness campaigns. In addition to the above considerations, several targeted and actionable strategies can further strengthen Nipah virus prevention and control efforts in Bangladesh.

1. Promotion of safe food practices related to date palm sap consumption.
2. Implementation of community awareness and behavioral change interventions.
3. Strengthening infection prevention and control (IPC) measures in healthcare settings.
4. Enhancement of surveillance and early outbreak detection systems.
5. Promotion of one health–based multisectoral collaboration.

## Supporting information

S1 Questionnaire Survey questionnaire on NiV infection risk. (DOCX)

S1 Table Selected divisions, districts, and upazilas in Bangladesh for a cross-sectional survey on Nipah virus infection risk, based on historical case occurrence and raw date-palm sap consumption patterns. (DOCX)

S2 Table Detailed participant responses to knowledge-based questions about Nipah virus. (DOCX)

S3 Table Participant attitudes toward Nipah virus infection and its control. (DOCX)

S4 Table Practice behaviors related to NiV prevention and control. (DOCX)
