# Supplementary material for: Community knowledge and response to Nipah virus infection and its transmission, prevention and control measures: Insights from a cross-sectional survey in Bangladesh
Source: PLoS Negl Trop Dis. 2025 Dec 17;19(12):e0013855. doi: 10.1371/journal.pntd.0013855 (PMC12725565; doi:10.1371/journal.pntd.0013855)
Supplement: S1 Questionnaire — (DOCX) [file pntd.0013855.s001.docx]

**S1 Questionnaire: Survey Questionnaire on Nipah Virus Infection Risk**

Section A: Sociodemographic Information

Name of Data Enumerator:

Participants Name:…………………………….. Location:…………………………………….

Mobile Number: ………………………Geographical Location (Lattitude, Longitude): ………………………

1. **Age**
   ☐ 13–18 years
   ☐ 18–25 years
   ☐ 26–35 years
   ☐ 36–45 years
   ☐ Above 45 years
2. **Gender**
   ☐ Male
   ☐ Female
3. **Education Level**
   ☐ No or Primary Education
   ☐ Secondary
   ☐ Higher Secondary
   ☐ Tertiary (Bachelor’s & above)
4. **Occupation**
   ☐ Student
   ☐ Homemaker
   ☐ Manual Labor & Unemployed
   ☐ Service Holder (Government/Private)
   ☐ Business/Self-employed
5. **Monthly Income**
   ☐ No income
   ☐ Below 10,000 BDT
   ☐ 10,001–30,000 BDT
   ☐ Above 30,000 BDT
6. **Type of Living Area**
   ☐ Peri-urban / Semi-urban
   ☐ Rural
7. **Distance from raw date palm sap sources**
   ☐ Less than 5 km
   ☐ 5–10 km
   ☐ More than 10 km
8. **Distance from nearest healthcare facility**
   ☐ 1–5 km
   ☐ 6–10 km
   ☐ More than 10 km

**Section B: Knowledge about Nipah Virus**

(Answer options: **Yes / Maybe / No**)

1. Do you usually drink raw date palm sap?
2. Have you heard of Nipah virus encephalitis?
3. Do you know how Nipah virus is transmitted to humans?
4. Can Nipah virus be transmitted through raw date palm sap?
5. Do fruit bats carry Nipah virus?
6. Do you know the symptoms of Nipah virus encephalitis?
7. Have you heard about any Nipah virus outbreaks in Bangladesh?
8. Do you know the case fatality rate of Nipah virus encephalitis?
9. Are you aware of any preventive measures for Nipah virus?
10. Do you know if Nipah virus can be transmitted from person to person?
11. Have you heard about the role of fruit bats in spreading Nipah virus?
12. Are you aware of the regions in Bangladesh where Nipah virus outbreaks are common?

**Section C: Attitudes towards Nipah Virus**

(Answer options: **Strongly agree / Agree / Neutral / Disagree / Strongly disagree**)

1. I believe that Nipah virus encephalitis is a serious health threat.
2. Drinking raw date palm sap is risky for Nipah virus infection.
3. The government should do more to control Nipah virus outbreaks.
4. Avoiding raw date palm sap can prevent Nipah virus infection.
5. I am willing to change the habit of consuming raw date palm sap to avoid Nipah virus infection.
6. I trust the health information on Nipah virus.
7. Public awareness campaigns about Nipah virus are effective.

**Section D: Practices related to Nipah Virus Prevention**

(Answer options: **Always / Often / Sometimes / Rarely / Never**)

1. How often do you avoid consuming raw date palm sap?
2. How often do you boil or treat raw date palm sap before drinking?
3. How often do you avoid visiting outbreak-prone areas during winter?
4. How often do you seek medical advice if you suspect Nipah virus symptoms?
5. How often do you participate in community health programs about Nipah virus?
6. How often do you share information about Nipah virus prevention with others?
7. How often do you follow the official guidelines for Nipah virus infection prevention?
8. I seek medical help if I have Nipah virus-like symptoms.
