# Supplementary material for: Community knowledge and response to Nipah virus infection and its transmission, prevention and control measures: Insights from a cross-sectional survey in Bangladesh
Source: PLoS Negl Trop Dis. 2025 Dec 17;19(12):e0013855. doi: 10.1371/journal.pntd.0013855 (PMC12725565; doi:10.1371/journal.pntd.0013855)
Supplement: S1 Table — (DOCX) [file pntd.0013855.s002.docx]

**S1 Table.** Selected divisions, districts, and upazilas with the latest (2022) population density (per km²) in Bangladesh for a cross-sectional survey on Nipah virus infection risk, based on historical case occurrence and raw date-palm sap consumption patterns.

| **Division (N=8)** | **District (N=16)** | **Area (km²)** | **Density (per km²)** | **Upazilas (Sub-district; N=48)** |
| --- | --- | --- | --- | --- |
| Dhaka | Faridpur | 2,052.86 | **1,054** | Bhanga, Faridpur Sadar, Alfadanga |
|  | Rajbari | ~1,092 | **1,089** | Rajbari Sadar, Pangsha, Baliakandi |
| Khulna | Meherpur | 741.62 | **951** | Meherpur Sadar, Mujibnagar, Gangni |
|  | Kushtia | 1,608.78 | **1,336** | Kushtia Sadar, Bheramara, Mirpur |
| Rajshahi | Naogaon | 3,435.67 | **810** | Naogaon Sadar, Patnitala, Dhamoirhat |
|  | Rajshahi | 2,407.01 | **1,164** | Rajshahi Sadar, Godagari, Bagha |
| Rangpur | Lalmonirhat | 1,247.37 | **1,145** | Hatibandha, Lalmonirhat Sadar, Aditmari |
|  | Thakurgaon | 1,842.60 | **868** | Thakurgaon Sadar, Baliadangi, Pirganj |
| Mymensingh | Mymensingh | 4,363.56 | **617** | Mymensingh Sadar, Trishal, Muktagacha |
|  | Jamalpur | ~2,032 | **1,231** | Jamalpur Sadar, Melandaha, Madarganj |
| Chattogram | Chattogram | ~5,280 | **1,736** | Fatikchhari, Satkania, Boalkhali |
|  | Cox’s Bazar | ~2,492 | **1,133** | Cox’s Bazar Sadar, Ramu, Ukhiya |
| Barishal | Pirojpur | 1,277.80 | **938** | Pirojpur Sadar, Nesarabad, Mathbaria |
|  | Barguna | 1,831.31 | **552** | Barguna Sadar, Amtali, Patharghata |
| Sylhet | Moulvibazar | 2,799.38 | **759** | Moulvibazar Sadar, Sreemangal, Barlekha |
|  | Sylhet | ~3,452 | **1,117** | Sylhet Sadar, Beanibazar, Golapganj |
