# Supplementary material for: Community knowledge and response to Nipah virus infection and its transmission, prevention and control measures: Insights from a cross-sectional survey in Bangladesh
Source: PLoS Negl Trop Dis. 2025 Dec 17;19(12):e0013855. doi: 10.1371/journal.pntd.0013855 (PMC12725565; doi:10.1371/journal.pntd.0013855)
Supplement: S2 Table — (DOCX) [file pntd.0013855.s003.docx]

| **S2 Table.** Detailed participant responses to knowledge-based questions about Nipah virus. | |
| --- | --- |
| **Characteristic** | **Frequency (%)** |
| **Have you heard of Nipah virus encephalitis?** |  |
| Yes | 332 (60.9%) |
| May be | 18 (3.3%) |
| No | 195 (35.8%) |
| **Do you know how Nipah virus is transmitted to humans?** |  |
| Yes | 349 (64.0%) |
| May be | 34 (6.2%) |
| No | 162 (29.7%) |
| **Can Nipah virus be transmitted through raw date palm sap ?** |  |
| Yes | 355 (65.1%) |
| May be | 86 (15.8%) |
| No | 104 (19.1%) |
| **Do fruit bats carry Nipah virus ?** |  |
| Yes | 361 (66.2%) |
| May be | 91 (16.7%) |
| No | 93 (17.1%) |
| **Do you know the symptoms of Nipah virus encephalitis?** |  |
| Yes | 105 (19.3%) |
| May be | 53 (9.7%) |
| No | 387 (71.0%) |
| **Have you heard about any Nipah virus outbreaks in Bangladesh?** |  |
| Yes | 235 (43.1%) |
| May be | 46 (8.4%) |
| No | 264 (48.4%) |
| **Do you know the case fatality rate of Nipah virus encephalitis?** |  |
| Yes | 62 (11.4%) |
| May be | 43 (7.9%) |
| No | 440 (80.7%) |
| **Are you aware of any preventive measures for Nipah virus?** |  |
| Yes | 157 (28.8%) |
| May be | 50 (9.2%) |
| No | 338 (62.0%) |
| **Do you know, Nipah virus can be transmitted from person to person?** |  |
| Yes | 148 (27.2%) |
| May be | 52 (9.5%) |
| No | 345 (63.3%) |
| **Have you heard about the role of fruit bats in spreading Nipah virus?** |  |
| Yes | 300 (55.0%) |
| May be | 39 (7.2%) |
| No | 206 (37.8%) |
| **Are you aware of the regions in Bangladesh where Nipah virus**  **outbreaks are common?** |  |
| Yes | 96 (17.6%) |
| May be | 47 (8.6%) |
| No | 402 (73.8%) |
