# Supplementary material for: Community knowledge and response to Nipah virus infection and its transmission, prevention and control measures: Insights from a cross-sectional survey in Bangladesh
Source: PLoS Negl Trop Dis. 2025 Dec 17;19(12):e0013855. doi: 10.1371/journal.pntd.0013855 (PMC12725565; doi:10.1371/journal.pntd.0013855)
Supplement: S3 Table — (DOCX) [file pntd.0013855.s004.docx]

| **S3 Table.** Participant attitudes toward Nipah virus infection and its control. |  |
| --- | --- |
| **Characteristic** | **Frequency (%)** |
| **I believe that Nipah virus encephalitis is a serious health threat.** |  |
| Strongly agree | 144 (26.4%) |
| Agree | 264 (48.4%) |
| Neutral | 122 (22.4%) |
| Disagree | 11 (2.0%) |
| Strongly disagree | 4 (0.7%) |
| **Drinking Raw date palm sap is risky for Nipah virus infection** |  |
| Strongly agree | 155 (28.4%) |
| Agree | 268 (49.2%) |
| Neutral | 114 (20.9%) |
| Disagree | 6 (1.1%) |
| Strongly disagree | 2 (0.4%) |
| **I feel that the government should do more to control Nipah virus outbreaks.** |  |
| Strongly agree | 175 (32.1%) |
| Agree | 297 (54.5%) |
| Neutral | 66 (12.1%) |
| Disagree | 5 (0.9%) |
| Strongly disagree | 2 (0.4%) |
| **I think that avoiding raw date palm sap can prevent Nipah virus infection** |  |
| Strongly agree | 110 (20.2%) |
| Agree | 271 (49.7%) |
| Neutral | 124 (22.8%) |
| Disagree | 33 (6.1%) |
| Strongly disagree | 7 (1.3%) |
| **I am willing to change the habits of consuming raw date palm sap to avoid nipah virus infection (If participant usually consume)** |  |
| Strongly agree | 96 (17.6%) |
| Agree | 259 (47.5%) |
| Neutral | 164 (30.1%) |
| Disagree | 18 (3.3%) |
| Strongly disagree | 8 (1.5%) |
| **I trust the health information on Nipah Virus** |  |
| Strongly agree | 120 (22.0%) |
| Agree | 377 (69.2%) |
| Neutral | 43 (7.9%) |
| Disagree | 5 (0.9%) |
| Strongly disagree | 0 (0.0%) |
| **Public awareness campaigns about Nipah virus are effective.** |  |
| Strongly agree | 193 (35.4%) |
| Agree | 318 (58.3%) |
| Neutral | 27 (5.0%) |
| Disagree | 7 (1.3%) |
| Strongly disagree | 0 (0.0%) |
