# Supplementary material for: Community knowledge and response to Nipah virus infection and its transmission, prevention and control measures: Insights from a cross-sectional survey in Bangladesh
Source: PLoS Negl Trop Dis. 2025 Dec 17;19(12):e0013855. doi: 10.1371/journal.pntd.0013855 (PMC12725565; doi:10.1371/journal.pntd.0013855)
Supplement: S4 Table — (DOCX) [file pntd.0013855.s005.docx]

| **S4 Table.** Practice behaviors related to NiV prevention and control. | |
| --- | --- |
| **Characteristic** | **Frequency (%)** |
| **How often do you avoid consuming raw date palm sap?** |  |
| Always | 156 (28.6%) |
| Often | 117 (21.5%) |
| Sometimes | 97 (17.8%) |
| Rarely | 103 (18.9%) |
| Never | 72 (13.2%) |
| **How often do you boil or treat raw date palm sap?** |  |
| Always | 96 (17.6%) |
| Often | 58 (10.6%) |
| Sometimes | 64 (11.7%) |
| Rarely | 90 (16.5%) |
| Never | 237 (43.5%) |
| **How often do you avoid outbreak known area during winter ?** |  |
| Always | 94 (17.2%) |
| Often | 60 (11.0%) |
| Sometimes | 65 (11.9%) |
| Rarely | 57 (10.5%) |
| Never | 269 (49.4%) |
| **How often do you seek medical advice if you suspect Nipah virus symptoms?** |  |
| Always | 149 (27.3%) |
| Often | 39 (7.2%) |
| Sometimes | 48 (8.8%) |
| Rarely | 46 (8.4%) |
| Never | 263 (48.3%) |
| **How often do you participate in community health programs about Nipah virus?** |  |
| Always | 65 (11.9%) |
| Often | 48 (8.8%) |
| Sometimes | 44 (8.1%) |
| Rarely | 76 (13.9%) |
| Never | 312 (57.2%) |
| **How often do you share information about Nipah virus prevention with others?** |  |
| Always | 91 (16.7%) |
| Often | 58 (10.6%) |
| Sometimes | 87 (16.0%) |
| Rarely | 101 (18.5%) |
| Never | 208 (38.2%) |
| **How often do you follow the guideline of Nipah virus infection ?** |  |
| Always | 107 (19.6%) |
| Often | 63 (11.6%) |
| Sometimes | 69 (12.7%) |
| Rarely | 70 (12.8%) |
| Never | 236 (43.3%) |
| **I seek medical help if i have nipah virus infection like symptoms** |  |
| Always | 282 (51.7%) |
| Often | 45 (8.3%) |
| Sometimes | 51 (9.4%) |
| Rarely | 41 (7.5%) |
| Never | 126 (23.1%) |
